# Patient perspective on the use of carbon fibre plates for extremity fracture fixation

**DOI:** 10.1007/s00590-023-03473-6

**Published:** 2023-01-19

**Authors:** Vasileios P. Giannoudis, Paul Rodham, Antony Antypas, Niki Mofori, George Chloros, Peter V. Giannoudis

**Affiliations:** 1 LIMM Section Musculoskeletal Disease, Academic Department of Trauma and Orthopaedics, School of Medicine, Leeds General Infirmary, University of Leeds, Clarendon Wing, Level A, Clarendon Way, Leeds, LS1 3EX UK; 2 Leeds General Infirmary, Health Education Yorkshire and Humber, Leeds, UK; 3 Athens, Greece; 4 Orthopaedic Surgeon, Athens, Greece

**Keywords:** Carbon fibre, Implant, PEEK, Fracture fixation

## Abstract

**Introduction:**

Carbon fibre-reinforced polyetheretherketone (CFR-PEEK) plates represent an exciting development within trauma and orthopaedic surgery, offering advantages including radiolucency, material properties similar to bone, and lack of localised tissue reaction. As more call for trials examining their use, there is no data available as to the acceptability of these implants to patients. This study aimed to therefore examine the acceptability of CFR-PEEK plates to patients undergoing fracture surgery.

**Methods:**

This was a prospective cross-sectional survey of patients undergoing surgery for a fracture of the ankle, distal femur, distal radius, or proximal humerus. Once a decision had been made to pursue operative fixation with a plate, patients were provided with descriptions of both CFR-PEEK and stainless steel and titanium metal implants alongside the current clinical evidence. All patients undertook a questionnaire examining their views as to the advantages and disadvantages of CFR-PEEK plates, and whether they would be happy to participate in a trial comparing both.

**Results:**

Ninety-nine patients were happy to participate (64 females, mean age 50). Eighty-seven patients reported that they would want a CFR-PEEK implant for their fracture, and 76 reported that they would be willing to participate in an RCT comparing their use. Commonly reported advantages included radiolucency, low weight and biocompatibility. Disadvantages reported included cost and concerns regarding durability.

**Conclusions:**

This study demonstrates that CFR-PEEK implants would be acceptable to patients undergoing fracture surgery, with high numbers of patients stating that they would be willing to participate in a randomised study examining their use.

**Supplementary Information:**

The online version contains supplementary material available at 10.1007/s00590-023-03473-6.

## Introduction

Carbon fibre-reinforced polyetheretherketone (CFR-PEEK) plates are an exciting prospect in the field of Orthopaedic surgery, possessing numerous advantageous including: radiolucency—enabling accurate fracture reduction and healing monitoring; a modulus of elasticity nearer to bone when compared to metal implants; decreased artefact in magnetic resonance imaging—allowing accurate assessment of co-existing soft tissue injuries following the management of fractures, and an absence of immune response to the implant that may result in localised inflammatory reactions [1, 2].

A recent systematic review found that whilst the utility of CFR-PEEK implants appears promising, there is little high level evidence to justify transition to their routine use [3]. Just two randomised controlled trials have been published examining the use of CFR-PEEK plates in the distal radius and proximal humerus. Both studies demonstrated equivalent healing times, clinical outcomes and patient reported outcomes; however, there were just 15 and 37 patients in the CFR-PEEK groups, respectively [4, 5]. Further prospective and retrospective observational studies have demonstrated very high rates of union, with excellent clinical outcomes and a low rate of complications [3].

Whilst the usage of CFR-PEEK implants is an exciting prospect, there is a requirement for further studies in the form of large randomised controlled trials to demonstrate their non-inferiority prior to routine adoption in extremity fracture fixation. Patient and public involvement (PPI) is a key feature of planning any large randomised controlled trial, and thus far there have been no studies examining the acceptability of these implants to the population at risk [6].

The aim of the presented study was therefore to examine the acceptability of CFR-PEEK implants compared to traditional metal implants in patients undergoing operative fixation of extremity fractures.

## Methods

We conducted a prospective cross-sectional survey of patients undergoing operative fixation of extremity fractures at our level one trauma centre. This study received Institutional review board approval (number #0258). Inclusion criteria were restricted to adult patients with fractures of the ankle, distal femur, distal radius and proximal humerus; given that these are the body areas where the use of these plates has been previously examined in the literature [3]. Exclusion criteria included patients undergoing non-operative management, patients with pathological fractures, patients 16 years and younger and patients who were not undergoing fixation with a plate.

**Fig. 1:**
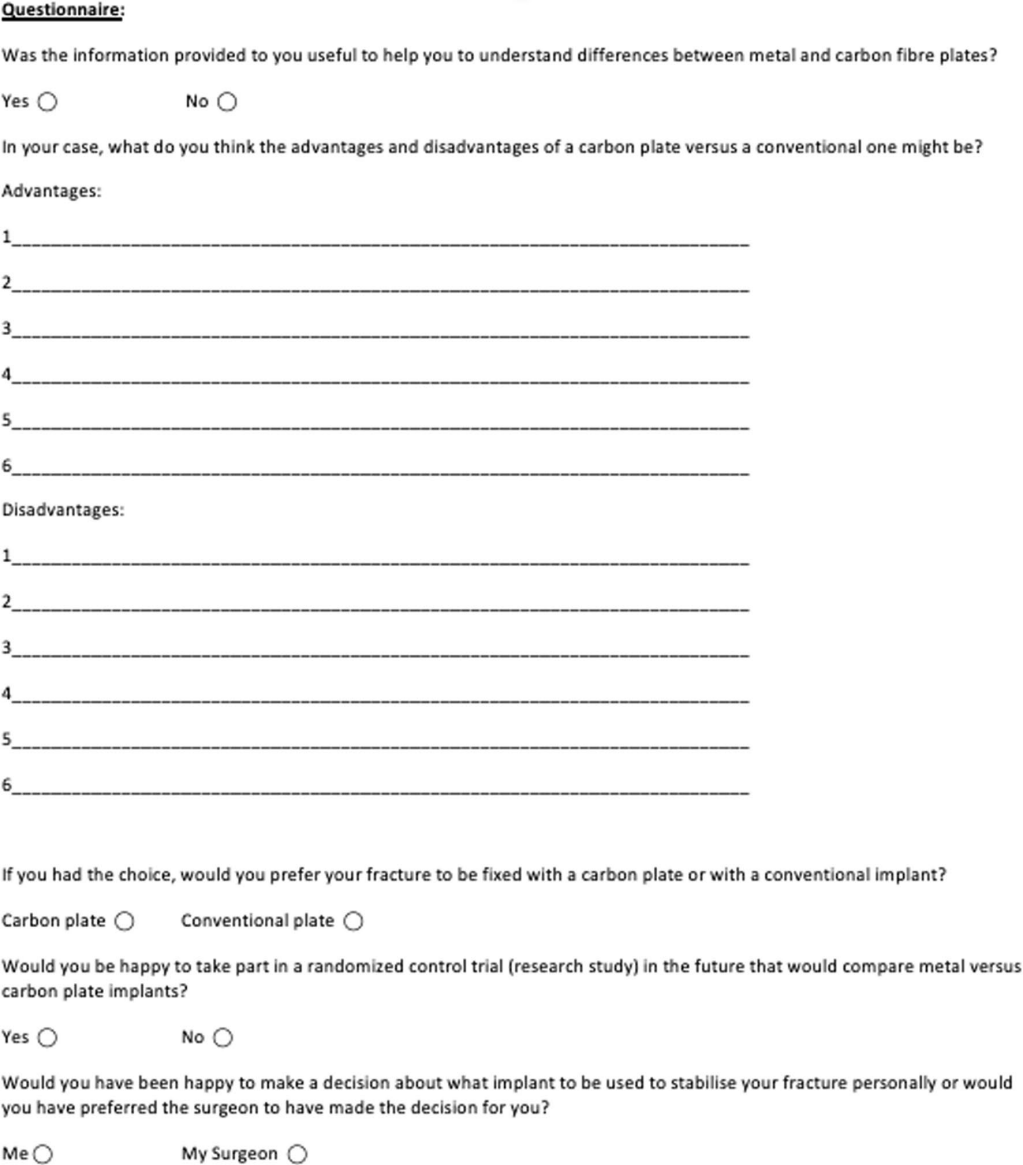
Questionnaire completed by patients following review of information leaflet

Patients were approached after a decision for open reduction and internal fixation (ORIF) had been made via a discussion between the patient and an Orthopaedic clinician who was independent to the study. Upon agreement to participate in the study patients were provided with an information leaflet summarising the current literature surrounding the implant in the body area they had injured. They were also provided with a table of summary of the advantages and disadvantages of the CFR-PEEK and traditional metal implants, alongside images of the fixation devices and a physical example of each device (Appendix 1). Following this they were provided with a questionnaire assessing their opinion on the perceived advantages and disadvantages of the two plating systems, whether they would be willing to receive a carbon implant instead of a metal implant, and whether they would have been willing to participate in an RCT comparing the use of CFR-PEEK plates with metal (stainless steel/titanium) plating systems (Fig. 1).

Basic descriptive data are presented demonstrating the patient population surveyed, and their responses to the questions posed. Data were subsequently analysed using SPSS (IBM, Chicago, Illinois, United States). Assumptions for parametric analysis were met and therefore continuous data were analysed using an unpaired two-tailed *T*-test, whilst categorical data were analysed using a Chi-square test and Fishers Exact test when *N* < 5.

## Results

Ninety-nine patients were happy to partake in the study and completed the questionnaire. This included 64 females and 35 males, with a mean age of 50 (range 18–95). Sixty patients had sustained a fracture to their ankle, twenty-four to their distal radius, ten to their proximal humerus, and five to their distal femur. All patients approached felt that the patient information leaflet provided them with sufficient information to understand the differences between the metal and CFR-PEEK plates. Following review of the information 86 patients indicated that they would prefer to have their injuries stabilised with a CFR-PEEK plate, with just 13 preferring the use of a conventional metal plate. Regarding entry into a potential RCT examining the difference between the two fixation devices, 76 patients stated that they would be happy to be recruited to participate, with just 23 opting against. Patients who stated that they were willing to participate in an RCT were noted to be significantly younger than those who would not (49 years old vs. 59 years old; *p* = 0.01). There was no difference in acceptability when comparing gender or site of injury. In terms of decision-making as to the ideal implant for their fixation, 70 patients stated that they would favour their surgeon made this decision, whilst 29 stated they would prefer to decide this once adequate information had been provided. This could not be predicted by age, gender nor site of injury.

Patients were provided with the opportunity to provide free text responses to their perceived advantages and disadvantages of the CFR-PEEK plating system, compared to traditional metal implants. When assessing advantages of this system, responses were received from 96 patients, with 43 patients reporting a single advantage, 50 reporting two advantages, and four reporting three advantages. The most common advantages noted were the reduced weight of the implants (*n* = 64), radiolucency (*n* = 52), and biocompatibility (*n* = 10). Responses regarding perceived advantages are summarised in Table 1. Regarding disadvantages (Table 2), responses were received from only 44 patients, with 35 reporting a single disadvantage, and nine reporting two disadvantages. The most common perceived disadvantages were cost (*n* = 26), distrust in a new technology (*n* = 16), and concerns regarding durability (*n* = 5).

**Table 1:** Patients perceived advantages of the CFR-PEEK plates

Theme	Number of responses
Lightweight material	64
Radiolucency	52
Biocompatibility	10
Strong material	7
New technology	5
Smaller size	5
Reduced infection	5
Long lifespan	2
MRI compatibility	1
Reduced complications	1
Reduced stress-shielding	1
Ease of removal	1
Flexible material	1

**Table 2:** Patients perceived disadvantages of the CFR-PEEK plates

Theme	Number of responses
Cost	26
New technology	15
Durability	5
Reduced resistance	4
Needs trialled	1
Radiolucency	1
Irritation	1

## Discussion

The use of carbon plates within the field of Orthopaedic trauma represents an exciting potential advancement within a field that is continually innovating [7]. There is a relative paucity of evidence regarding the clinical use of carbon-based implants, with just two small RCTs available at present. In order to progress this technique there would therefore need to be further large RCTs, which if they were to demonstrate equivalence would allow surgeons to take further confidence in the use of carbon plates to reconstruct fractures.

As medicine moves towards a more patient-centred and evidence-based approach, the involvement of patients early in the process of devising research is critical [8]. Involvement of patients early in the research process has been demonstrated to improve engagement, often leading to revision of the assessment type once the research group have established what outcomes are important to the patient cohort at risk [9, 10].

In this study, we aimed to assess patients’ perceptions in the United Kingdom about the use of Carbon implants in the operative management of extremity trauma. Our study has demonstrated that if patients were to sustain a fracture, 87% would be happy to receive a carbon-fibre placed implant. Furthermore, from our patient cohort 77% would be happy to participate in a trial examining the use of carbon implants, compared to the current standard metal implants.

There were a number of recurrent positive themes expressed by our patient cohort, including the lightweight nature of the implant, the radiolucency, and the biocompatibility. There were mixed opinions as to the use of new technology, with 15 patients reporting this as a potential disadvantage of the implant, whereas five saw this as an advantage. This is frequently the case with the adoption of a new technology; however, the overall response with regards to participate was positive. Not only is it important to assess the opinions of patients to new technology, but the equipoise must be established within the community providing this treatment, with uptake from surgeons engaging in such a trial often lower than that of the patient population [11, 12].

There were a number of perceived disadvantages as to the new implant. In particular there was concerns regarding the cost of the implementation of a new technology, and the potential durability. In recruiting to a trial fears regarding durability could be allayed with current biomechanical evidence suggesting that carbon plates have peak failure energies and lower rates of permanent deformation compared to stainless steel implants [13]. Compared to titanium implants, carbon implants have again been demonstrated to have similar strength in axial, bending and torsional deformation; though with lower stiffness [14]. The Young’s modulus of CFR-PEEK implants can be modulated through alteration of the carbon fibre and is actually closer to that of cortical bone when compared to titanium or steel implants [15]. This may produce benefits in terms of generating the optimal fracture site strain, and reduce complications associated with excessively stiff materials such as stress shielding [16].

## Conclusion

The findings from this study examining the patient acceptability of CFR-PEEK implants are positive and highlight a significant acceptability from patients regarding potential recruitment to a randomised trial. Counselling during such a trial should focus on allaying fears regarding strength and durability of these implants, in line with the current literature.

## Supplementary Information

Below is the link to the electronic supplementary material.

Supplementary file1 (DOCX 1372 KB)
