# Supplementary material for: Patient perspective on the use of carbon fibre plates for extremity fracture fixation
Source: Eur J Orthop Surg Traumatol. 2023 Jan 19;33(6):2573–7. doi: 10.1007/s00590-023-03473-6 (PMC10368544; doi:10.1007/s00590-023-03473-6)

**Appendix 1** – Patient information leaflets provided summarising current literature surrounding use of CFR-PEEK in a) proximal humerus, b) distal radius, c) distal femur, d) ankle


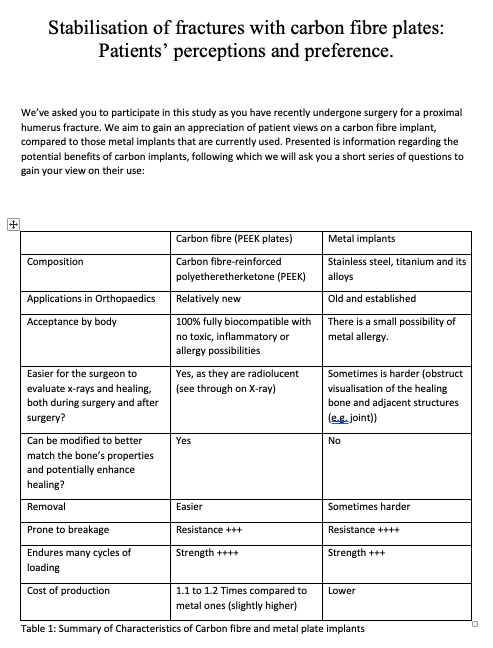

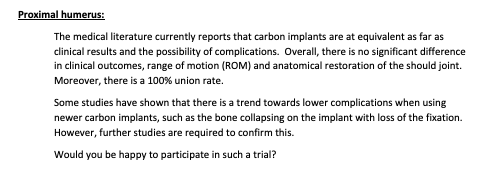


a

b


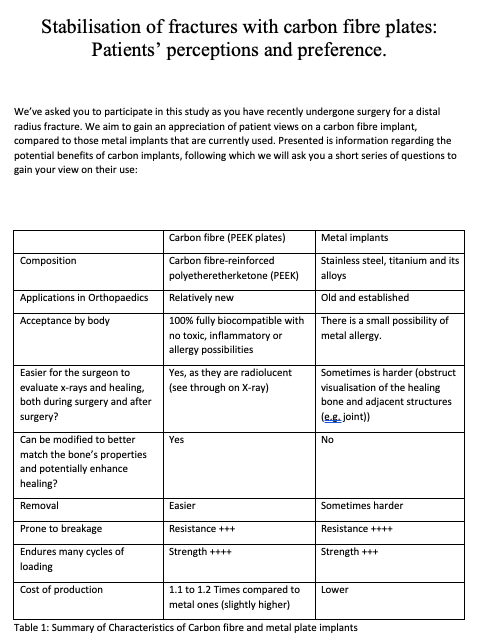

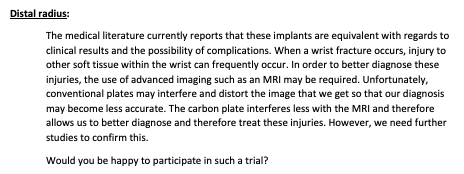


c


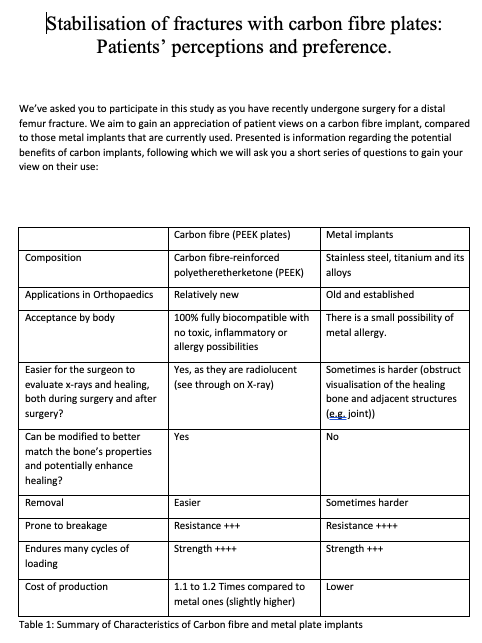

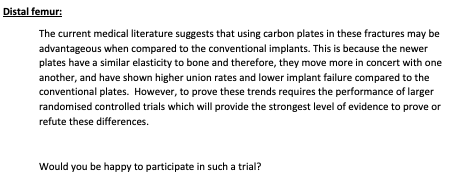


d


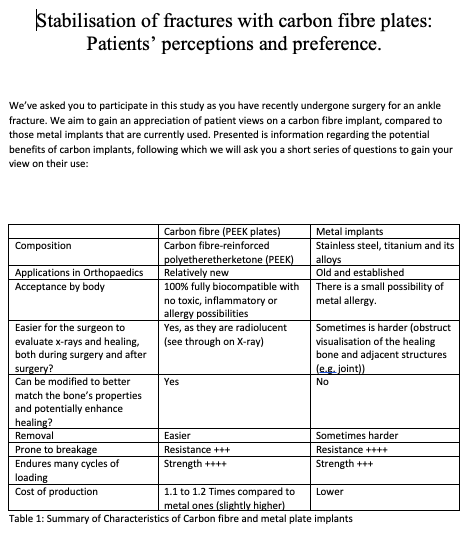

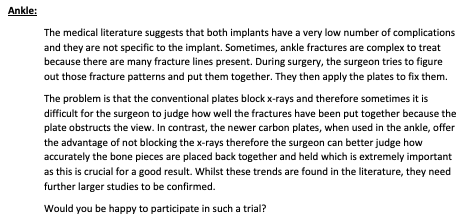

Supplement: Supplementary file 1 — Supplementary file1 (DOCX 1372 KB) [file 590_2023_3473_MOESM1_ESM.docx]
